# Electrophysiological Profiling of Exocytosis during Early-Stage Development of the Zebrafish Lateral Line

**DOI:** 10.1523/ENEURO.0479-25.2026

**Published:** 2026-09-17

**Authors:** Jiali Wang, Ane Landajuela, Joseph Santos-Sacchi, Erdem Karatekin, David Zenisek

**Affiliations:** ^1^Cellular and Molecular Physiology, Yale University, New Haven, Connecticut 06510; ^2^Nanobiology Institute, Yale University, West Haven, Connecticut 06477; ^3^Surgery (Otolaryngology), Yale University, New Haven, Connecticut 06510; ^4^Neuroscience, Yale University, New Haven, Connecticut 06510; ^5^Molecular Biophysics and Biochemistry, Yale University, New Haven, Connecticut 06511; ^6^Wu Tsai Institute, Yale University, New Haven, Connecticut 06510; ^7^SPPIN - Saints-Pères Paris Institute for the Neurosciences, Centre National de la Recherche Scientifique (CNRS), Université Paris Cité, Paris F-75006, France; ^8^Ophthalmology and Visual Sciences, Yale University, New Haven, Connecticut 06510

**Keywords:** developmental biology, hair cell, otoferlin, ribbon synapse, synapse, vestibular system

## Abstract

Hair cells of the zebrafish lateral line have proven to be a good model for studying hair cell function in a system that is easily genetically manipulated, rapidly develops, and is experimentally accessible. However, characterization of potential developmental changes and possible differences along lateral line position are lacking. Here, we used in vivo patch clamp to investigate the exocytic properties of neuromast hair cells over early development across body location. Long depolarizations led to steady increases in membrane capacitance, presumably due to exocytosis of vesicles localized to ribbon synapses. The magnitude and kinetics of capacitance changes did not vary significantly across the L1–L6 positions of neuromasts along the lateral line, but the magnitudes were found to be significantly smaller in hair cells found in the tail region across all developmental time points. For each region, we found no significant changes in capacitance responses between 3 and 7 d after fertilization. Hair cell capacitance responses were greatly reduced in animals injected with CRISPR/Cas9 with gRNAs targeted to *otoferlin b*. These results confirm the essential role of otoferlin b in neuromast hair cell function, and they demonstrate the fidelity of CRISPR/Cas9 to rapidly mediate genetic removal of critical genes to study their impact on synaptic release.

## Significance Statement

The zebrafish lateral line is a well-established model system for studying hair cell function. In our manuscript, we systematically investigate these properties and find that properties remain consistent across developmental time, despite ongoing maturation of the synapse and discover key differences in hair cell responses in the tail region relative to those along the body. Moreover, we establish that disruption of *otoferlin b* by CRISPR/Cas9 dramatically reduces exocytosis. These studies provide important new information that are important for establishing a baseline for investigating the impact of genetic manipulations on hair cell function.

## Introduction

Hair cells play an important role in both the auditory and vestibular systems. In fish, lateral line hair cells—located in mechanosensory organs called neuromasts along the body—are functional analogs to mammalian hair cells. These neuromasts detect water flow and are essential for survival behaviors such as prey detection, mating, and predator avoidance (McHenry et al., 2009). Due to their structural and functional similarities to mammalian hair cells, the zebrafish lateral line system provides an excellent model for studying synaptic transmission, hair cell regeneration, and hearing-related mechanisms (Lenz and Avraham, 2011).

A key feature of hair cells is the synaptic ribbon, an electron-dense structure at the presynaptic active zone that tethers synaptic vesicles (SVs). Synaptic ribbons are specific to nonspiking cells of the auditory, vestibular, and visual systems that encode sensory information as graded changes in neurotransmitter release (Matthews and Fuchs, 2010). In hair cells, ribbons facilitate rapid and phase-locked exocytosis and signal transmission upon mechanical stimulation. While ribbon synapses share most proteins with conventional synapses, some proteins are uniquely expressed in all ribbon synapses or subsets of ribbon synapses (Thoreson and Zenisek, 2024). Among the proteins unique to hair cell ribbon synapses is the protein otoferlin, which is required for normal exocytosis in adult vertebrates (Roux et al., 2006; Reisinger et al., 2011; Moser et al., 2020).

The lateral line develops early in zebrafish embryogenesis. By 48 h postfertilization (hpf), a migrating primordium places clusters of cells, each forming a neuromast (L1–L6), with additional terminal neuromasts developing at the tail tip. Intercalary neuromasts later arise from proliferating interneuromast cells (Grant et al., 2005; Lopez-Schier and Hudspeth, 2005; Nunez et al., 2009). The lateral line develops rapidly in zebrafish, with hair cells emerging at 48 hpf and becoming capable of responding to sensory stimuli within 48 hpf (Metcalfe, 1985; Raible and Kruse, 2000; Lopez-Schier et al., 2004; Ma and Raible, 2009; Nunez et al., 2009; Trapani and Nicolson, 2010, 2011; Pujol-Marti and Lopez-Schier, 2013; Venkataraman et al., 2025) and reaching maturity by 5 d postfertilization (dpf; Kindt et al., 2012; Zhang and Kindt, 2022). Mature hair cells in zebrafish contain ∼130 vesicles per ribbon and 2–5 ribbons per lateral line hair cell (Obholzer et al., 2008; Lv et al., 2016; Sheets et al., 2017; David et al., 2024). However, functional heterogeneity among neuromasts remains unclear. A comprehensive electrophysiological analysis of synaptic function across neuromasts is lacking, and the timing of electrophysiological maturation has not been fully characterized. Previous studies have focused on differences between larval and juvenile fish (Olt et al., 2014, 2016) or at specific time points of development (Ricci et al., 2013; Lv et al., 2016); we focus here specifically on the changes over early time points in development.

In this study, we employed in vivo whole-cell electrophysiology to investigate the magnitude of calcium currents and exocytosis in lateral line hair cells during early development (3–7 dpf) and across different neuromasts. After blocking K^+^ currents with intracellular CsCl, whole-cell currents recorded using an *I*–*V* ramp protocol revealed a voltage-dependent inward current ranging from ∼10 to 15 pA. This inward current was previously identified as a Ca^2+^ current mediated by CaV1.3 channels expressed in lateral line hair cells (Santos-Sacchi, 2004; Ricci et al., 2013). We additionally tested the role of otoferlin, a critical protein in hair cell synaptic vesicle release (Roux et al., 2006; Reisinger et al., 2011; Moser et al., 2020) Zebrafish have two otoferlin genes, designated *otoferlin a* and *otoferlin b*. *otoferlin b* (*otofb*) has been shown to be the dominant isoform in neuromasts (Chatterjee et al., 2015). By using CRISPR-injections, we found significant impairments in exocytosis in *otof* crispants that did not vary across developmental time. Thus, unlike in mammalian inner hair cells where otoferlin expression gradually replaces that of synaptotagmin-1 within the initial 14 d after birth (Reisinger et al., 2011), in zebrafish, otof b expression is present around the time of synapse formation and is necessary for exocytosis even early in development(Chatterjee et al., 2015; Farrell et al., 2018). Our results further establish that CRISPR/Cas9 injections can be used in acute studies to monitor effects of protein deletion electrophysiologically at the single-cell level. Interestingly, we identified variations in function between neuromasts, particularly those at the tail in comparison with the body. Finally, we combined immunostaining of pre- and postsynaptic markers and analysis of otoferlin expression to assess hair cell functionality. We found that during 3–7 dpf, hair cell numbers increase, and synapses mature, but release properties of individual hair cells change little. These results highlight functional diversity among neuromasts, provide a baseline for future studies, and underscore the zebrafish lateral line as a valuable model for synaptic physiology and hearing research.

## Materials and Methods

### Zebrafish husbandry

Zebrafish were kept in accordance with the Yale University Animal Care and Use Committee guidelines. The *otofb* crispant has been generated by the CRISPR-Cas9 gene editing method. CRISPR-Cas9 protein (Integrated DNA Technologies) and gRNA were injected into WT zebrafish embryo at the one-cell stage. The gRNAs targeting *otofb* were selected from CHOPCHOP and produced by Integrated DNA Technologies. The gRNAs used, TCGGCCTGCAGGTAGTCGAAAGG and GACCTGAATCGATTTCCACGAGG, target exons 39 and 40 of otoferlin b, respectively. Genotyping was used to confirm injection efficiency by using standard PCR followed by Sanger sequencing. *otofb* crispants were compared with wild type as stated in the text.

### Zebrafish hair cell electrophysiology

Zebrafish of either sex ranging in age from 3 to 7 dpf were anesthetized with tricaine and mounted by stainless steel pins (Fine Science Tools: 26002-10) in a recording chamber. An upright Olympus microscope was used for viewing, and recordings were made with an Axon 200B amplifier with an Axon DD1322 digitizer, following previous work (Ricci et al., 2013). All recordings were made with the jClamp software (Scisoft). Hair cells for whole-cell patch clamp were selected at random locations within each neuromast. Cells were held at a resting potential of −60 mV. The extracellular solution contained the following (in mM): 125 NaCl, 1.0 KCl, 2.2 MgCl_2_, 2.8 CaCl2, 10 HEPES, and 6 d-glucose, 285 mOsm, pH 7.6. The pipette solution was composed of the following (in mM): 90 CsCl, 20 TEA, 5 Na_2_ATP, 3.5 MgCl_2_, 10 HEPES, and 1 EGTA, 260 mOsm, pH 7.2. Pipette resistance was typically 4–5 MΩ with Cs pipette solutions. Patch pipettes were pulled from 1.5-mm-outside-diameter thick-walled borosilicate glass (World Precision Instruments,TW150F-3) and coated with wax (Sybron Dental Specialties, #000625). Capacitance measurements were made with a dual-sine admittance technique (Santos-Sacchi, 2004). ΔCm was calculated as the difference between the final capacitance measurement taken after a 5 s depolarization and the baseline capacitance value recorded while the hair cell was held at −70 mV. With the dual-sine stimulus superimposed on command protocol waveforms, Ca^2+^ currents were measured with a rapid 500 ms voltage ramp from −80 to +40 mV. As mentioned in the Results section, no Na^+^ currents were observed, and currents recorded represent the activity of L-type Ca^2+^ channels (3, 20, 29, 30). Presumptive supporting cells were excluded from analysis, which we defined as cells with a membrane capacitance larger than 1.6 pF and no detectable Ca²^+^ currents. Recordings were made at room temperature. Data are reported as mean ± SEM. Electrophysiological data failed both the Lilliefors and Jarque–Bera tests for normality. Mann–Whitney or Kruskal–Wallis tests were performed to determine whether differences between group means were statistically significant. To estimate the rate of release, the capacitance changes during individual depolarizations were fit to a single exponential function: $C=A(1-\exp\;(-(t-t_{0})/\tau ))+b$ using the Origin software.

### Immunofluorescence

Zebrafish hair cell staining was performed as previously published (Lv et al., 2012). Briefly, 3–7 dpf zebrafish larvae were anesthetized and fixed in 4% paraformaldehyde (PFA) in PBS for 6 h. Next, fish were transferred to blocking buffer consisting of 2% bovine serum albumin, 3% normal goat serum, and 0.1% Triton X-100 in PBS, followed by incubating overnight with primary antibodies diluted in blocking buffer at 4°C. The samples were washed three times with PBS. They were then incubated with secondary antibodies for 4 h at room temperature, followed by additional washes. Finally, the samples were mounted and prepared for imaging.

The primary antibodies used for anti-otoferlin, selected from Picoband (PB9616, Boster Biological Technology) and pan-MAGUK (Neuromab), were diluted 1:1,000 and 1:500, respectively. CtBP Antibody (B-3) was diluted 1:1,000 (sc-55502, Santa Cruz Biotechnology). Both the pan-MAGUK and CtBP antibodies have been used extensively in zebrafish for localizing ribbons and afferent dendrites (Santos et al., 2006; Sheets et al., 2011, 2012, 2017; Lv et al., 2016; Dasgupta et al., 2024). The secondary antibodies [Alexa Fluor 488/643 goat anti-mouse/rabbit IgG(H + L), Molecular Probes] were diluted 1:200. The secondary antibodies (Alexa Fluor 647 goat anti-rabbit IgG1, Molecular Probes; Alexa Fluor 488 goat anti-rabbit IgG2a, Molecular Probes) were diluted 1:200.

Confocal images were acquired with a Zeiss LSM 950 laser-scanning confocal microscope using a Plan-Apochromat 63×/1.40 Oil DIC M27 objective. Digital images were processed and analyzed in ImageJ. For quantifying image intensity of otoferlin staining, the following parameters were used for all experiments: detector gain at 700 V for 488 mm wavelength, 1% laser intensity, and 650 V for 405 mm wavelength, 2% intensity. Image capture size was set to 1,024 × 1,024.

For HEK-293T immunostaining, cells were fixed with 4% PFA in PBS for 10–20 min at room temperature and subsequently processed for immunofluorescence under permeabilizing conditions. Primary antibodies were used at a dilution of 1:1,000, followed by the appropriate Alexa Fluor 568-conjugated secondary antibodies at a 1:2,000 dilution.

### Staining analysis

Quantitative image analysis was performed on raw images using the ImageJ (Fiji) software. For analysis, *z*-stacks collapsed into single *z*-projections with max intensity. Analysis of colocalization was carried out using the spots colocalization plugin. Estimated particle size and intensity threshold were set individually to maintain consistency. An entire neuromast was set as an ROI; particles were set as ovals to be detected within the ROI.

To quantitatively measure immunolabel density, we used identical imaging settings to compare the 3–7 dpf. Intensity, number, and locations of CtBP and MAGUK ROI were extracted from ImageJ and organized in Excel and plotted in Origin, GraphPad, or Excel.

As with our electrophysiology data, our fluorescence data for each comparison failed normality tests. All *p* values were measured using nonparametric tests: Mann–Whitney for single comparisons and Kruskal–Wallis for multiple comparisons.

## Results

### Electrophysiological characterization of zebrafish neuromast hair cells across body locations during development

To study hair cells of neuromasts, we used whole-cell patch clamp in live anesthetized zebrafish, as described previously (Ricci et al., 2013). The procedure involves perforating the neuromast periphery with a large-bore pipette to allow access of a patch pipette to the hair cell membrane. After achieving whole-cell configuration, the membrane capacitance *C_m_*, reflecting cell membrane area (Neher and Marty, 1982; Gillis, 1995; Sakmann and Neher, 1995), was continuously recorded. We used a high-frequency dual-sine approach that allows one to track and record capacitance despite the presence of membrane currents (Santos-Sacchi, 2004; Lv et al., 2016). For all recordings, we used a Cs^+^-based internal solution to block K^+^ channels. We found no evidence of transient Na^+^ currents in our experiments, which suggests a lack of voltage-gated Na^+^ channels. This contrasts with developing mouse hair cells, which do exhibit these channels and action potentials in immature hair cells (Marcotti et al., 2003). Since hair cells are typically known to lack other inward conductances (Moser and Beutner, 2000; Ricci et al., 2013; Li et al., 2014; Castellano-Munoz et al., 2016), the remaining current was presumed to reflect current from the L-type Ca^2+^ channels that drive neurotransmitter release (Sheets et al., 2012; Ricci et al., 2013; Lv et al., 2016).

**Figure 1.**
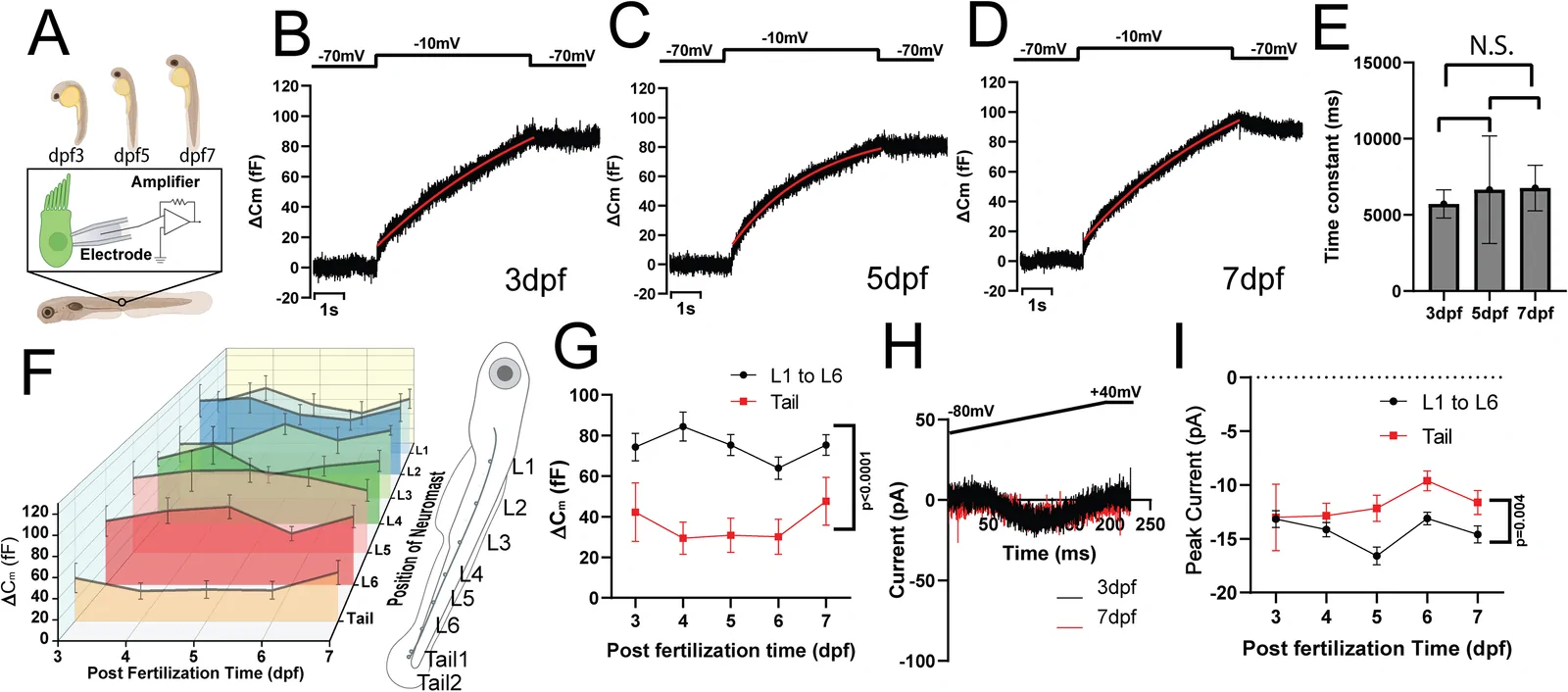
Exocytosis in neuromast hair cells across locations and developmental time. ***A***, Diagram of zebrafish development from 3 to 7 dpf and illustration of whole-cell recordings. ***B–D***, Capacitance responses to 5 s depolarizations to −10 mV in zebrafish neuromasts from 3 to 7 dpf taken from L1, L3, and L3, respectively. Corresponding best fits to the expression $C=A(1-\exp\;(-(t-t_{0})/\tau ))+b$ are shown. ***E***, The averaged time constants τ are shown for 3, 5, and 7 dpf. ***F***, Changes in capacitance $(\Delta C_{m})$ in response to 5 s depolarizations at different developmental times and neuromast positions. The neuromasts at the tail consistently showed smaller changes than the body (L1–L6). Since we observed a similar level of $\Delta C_{m}$ from L1 to L6, we combined these together as one group as plotted in ***G*** and ***I***. ***G***, The same dataset as in ***E***, but comparing capacitance changes averaged across neuromasts in L1–L6 to those at the tail at different development points (*p* < 0.0001). ***H***, Example of current measurements during a voltage ramp from −80 to +40 mV. ***I***, The negative peak currents from *I*–*V* ramp recordings as in ***H*** (*p* = 0.004). Error bars indicate SEM.

The posterior lateral line forms between 19 and 25 hpf (Ma and Raible, 2009; Sarrazin et al., 2010; Kindt et al., 2012). It contains several “primary” neuromasts, aligned along the anteroposterior body axis, labeled L1 to L6. Additionally, three neuromasts, labeled LII1 to LII3, develop outside the lateral line and are referred to as “secondary neuromasts” (Ghysen and Dambly-Chaudiere, 2007; Ma and Raible, 2009). These neuromasts are sensitive to water movements along perpendicular axes (Chou et al., 2017; Kindt and Sheets, 2018). Two additional neuromasts are found on the tail. In each neuromast, hair cells develop in pairs, their stereocilia facing opposite directions, enabling detection of water flow in both directions (Lopez-Schier et al., 2004; Nagiel et al., 2008; Faucherre et al., 2009; Chou et al., 2017; Lozano-Ortega et al., 2018). By 48 hpf, the embryonic lateral line pattern is fully established, but hair cells continue to develop over the next several days (Kindt et al., 2012; Suli et al., 2012; Zhang and Kindt, 2022). However, how electrophysiological properties vary across different neuromast locations and across developmental time is not fully understood. To address this question, we recorded from zebrafish between 3 and 7 dpf in neuromasts across the body and tail and subjected hair cells to 5 s depolarizations to −10 mV while monitoring *C_m_* as an index of exocytosis (Fig. 1*A*). Average capacitance traces of hair cells at the L1 or L3 positions at different developmental timepoints are shown in Figure 1*B*–*D*. Capacitance rises with depolarization in an exponentially slowing pattern as previously described (Ricci et al., 2013; Lv et al., 2016). To analyze the kinetics of exocytosis, we individually fit each trace to a single exponential function and averaged across hair cells for each developmental time point and found no difference (Fig. 1*E*).

We systematically recorded from L1 to L6 and tail one and tail two neuromasts from 3 to 7 dpf. A three-dimensional plot summarizes hair cell capacitance responses to 5 s depolarizations as a function of developmental time (dpf) and neuromast location (Fig. 1*F*). Quantification of capacitance changes showed little variation over developmental time whether in the body or the tail regions. For example, the mean Δ*C_m_* for L3 hair cells was 65.9 ± 21.4 fF at 3 dpf, 66.5 ± 18.4 fF at 4 dpf, and 82.3 ± 5.7 fF at 7 dpf (Fig. 1*F*). Statistical analysis confirmed no significant differences across the different time points or either analysis on L1–L6 or tail locations (*p* > 0.05).

We also found that the average capacitance response was largely consistent across neuromasts L1–L6 at all developmental stages. For example, following a 5 s depolarization at 7 dpf, the mean capacitance response Δ*C_m_* was 72.8 ± 12.6 fF for L1, 85.5 ± 17.6 for L2, 82.3 ± 5.7 fF for L3, 73.1 ± 12.3 for L4, 68.7 ± 11.2 for L5, and 71.1 ± 11.5 fF for L6, with no statistical difference among the measurements (*p* > 0.05).

Recordings from hair cells located on the tail neuromasts showed little variation over time as mentioned, but their capacitance responses were consistently smaller than those in the L1–L6 neuromasts. Since responses varied little from L1 to L6, we grouped responses from these neuromasts for subsequent analysis, as shown in Figure 1*G*. Capacitance responses were significantly smaller in tail hair cells than those in the body across all developmental time points measured (*p* < 0.0001). We next compared calcium currents elicited by 200 ms depolarizations over developmental time beginning at 3 dpf and continuing until 7 dpf. Example current recordings of hair cells subjected to 200 ms ramps from −80 to 40 mV for a 3 dpf (black) and 7 dpf (red) hair cells are shown in Figure 1*H*. In comparison, negative peak currents were similar at 3 and 4 dpf (*p* = 0.48 for 3 dpf, *p* = 0.18 for 4 dpf) for locations at the tail compared with L1–L6 but had smaller amplitude at later time points (Fig.1*I*). Thus, the lower magnitude of Ca^2+^ currents on the tail neuromasts may contribute to their smaller capacitance responses compared with L1–L6 neuromasts for 5–7 dpf.

### Maturation of hair cell synapses during early development

**Figure 2.**
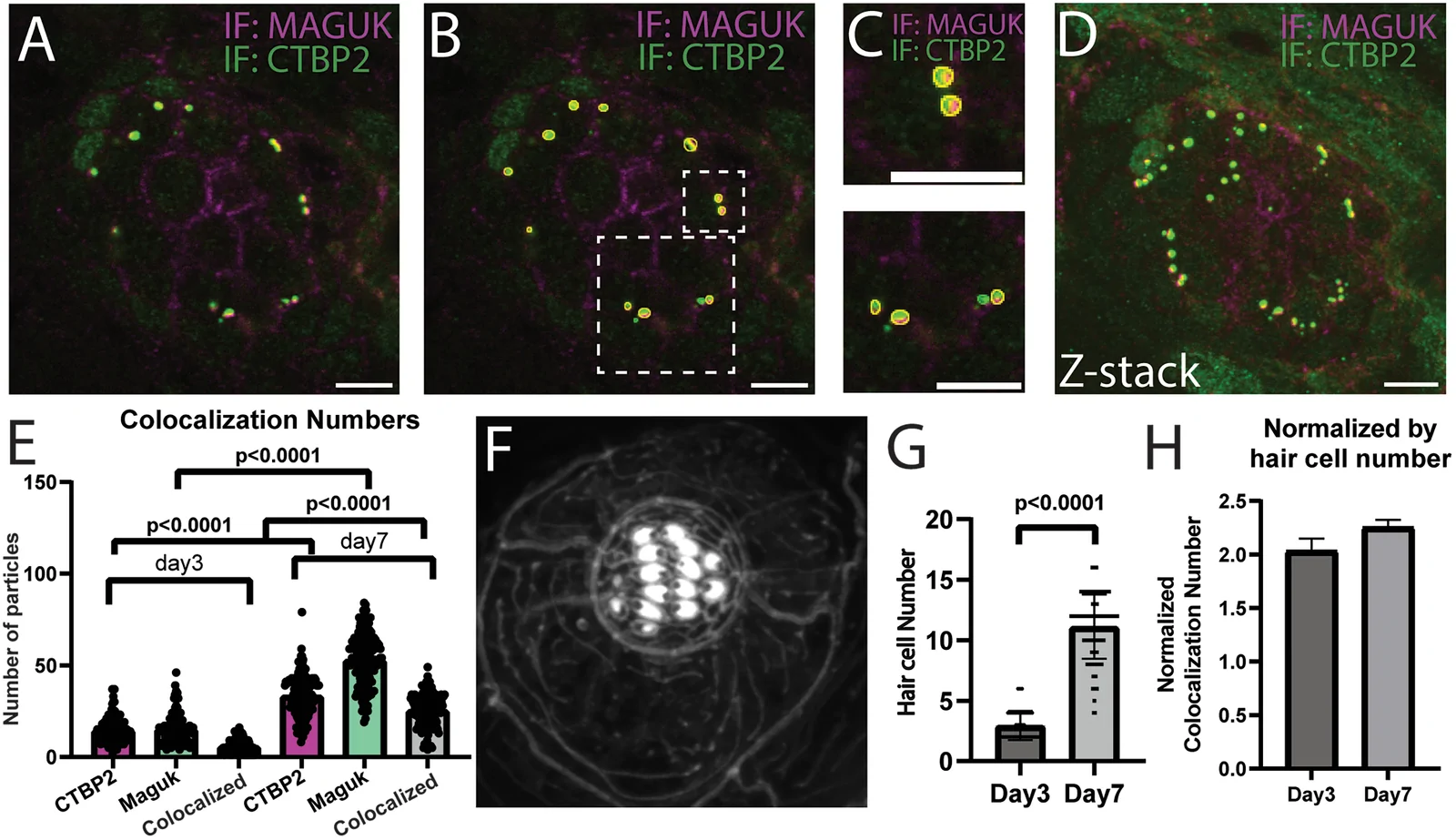
Synapse formation from Day 3 to Day 7. ***A***, Immunostaining of CTBP2 (Green) and MAGUK (magenta) in 3 dpf zebrafish. ***B***, The same image as in ***A*** but with assembled synapses marked by a yellow outline. Assembled synapses are defined by pre- and postsynaptic markers, CTBP2 and MAGUK, respectively, colocalized within 0.5 µm. Postsynaptic markers (magenta) are lacking near some presynaptic markers (green), indicating some synapses are yet to be assembled. ***C***, Magnified view of regions in ***B***. ***D***, *Z*-projection from the same neuromast shown in ***A–C***, *Z*-stack view of pre- (CTBP2, green) and postsynaptic markers (MAGUK, magenta) taken from 48 sections over 12.69 µm total distance. ***F***, A confocal microscopy *z*-stack projection of a neuromast, viewed from the top. The neuromast was from 7 dpf larva, at Position L1. Hair cell numbers were evaluated from cilia staining by fluorescent phalloidin. ***E***, The number of pre- and post- synaptic marker spots and their colocalization statistics, calculated from *z*-stack images as in D, using ImageJ (Schneider et al., 2012), shown for 3 and 7 dpf animals. This analysis included 112 neuromasts at 3 dpf and 164 neuromasts at 7 dpf. ***G***, The number of hair cells per neuromast. There were 3.0 ± 0.2 and 11.6 ± 0.2 hair cells per neuromast at 3 and 7 dpf, respectively. ***H***, The number of colocalized CTBP2/pan-MAGUK spots per neuromast, normalized to the number of hair cells. The average number of colocalized spots per hair cell was 2.03 ± 0.12 at 3 dpf and 2.25 ± 0.1 at 7 dpf. (*p* = 0.1).

Since we found that exocytosis was similar between 3 and 7 dpf for a given neuromast but changed based on location, we next investigated whether hair cell and synapse numbers also changed across location and time. To do so, we immunostained for the synaptic ribbon protein Ribeye and the postsynaptic marker pan-MAGUK, as done previously by other investigators (Lv et al., 2016; Sheets et al., 2017; Dasgupta et al., 2024; Fig. 2*A*–*D*). Ribeye is a gene product arising from an alternate start site to the transcriptional corepressor CtBP2 (Schmitz et al., 2000) and thus can be immunostained using antibodies that recognize CtBP2. We defined assembled ribbon synapses as those that contained both presynaptic ribbons (CtBP2) and postsynaptic (pan-MAGUK) markers in close proximity, while immature or ribbon precursors (David et al., 2024; Voorn et al., 2024) were defined as Ribeye spots that lacked nearby (<0.5 µm) postsynaptic markers (Nicolson, 2015; Akiba et al., 2019). The number of Ribeye spots, pan-MAGUK spots, and colocalized pan-MAGUK/Ribeye spots increased from Day 3 to Day 7. The number of colocalized markers was nearly identical in both the body and tail at 3 dpf (body 5.9 ± 0.4; tail 6.0 ± 0.6, *p* = 0.279) and 7 dpf (body 25.1 ± 0.9; tail 24.1 ± 1.3, *p* = 0.06), so we combined the data for presentation in Figure 2*E*.

Hair cell numbers also increase over this period of development (Harris et al., 2003; Hardy et al., 2021; Coffin et al., 2024), which may partially or completely account for the change in ribbon numbers. To account for hair cell proliferation, we normalized the number of colocalized spots to the number of cells in each neuromast. To identify hair cells, we used phalloidin to visualize hair cell bundles (Fig. 2*F*; Lopez-Schier et al., 2004) at the apical tip of hair cells and counted the number of hair bundles in each neuromast at 3 and 7 dpf (Fig. 2*G*). The number of hair cells per neuromast was consistent across the two locations at Day 3 (body 3.0 ± 0.2, tail 2.8 ± 0.4) and Day 7 (body 10.9 ± 0.3, tail 11.9 ± 0.4). The data combined across locations are presented in Figure 2*G*. When normalized to cell count at each developmental stage, there was no difference in the number of spots containing adjacent pre- and postsynaptic markers, as shown in Figure 2*H* (2.03 ± 0.12 at 3 dpf; 2.25 ± 0.1 at 7 dpf, *p* = 0.1).

**Figure 3.**
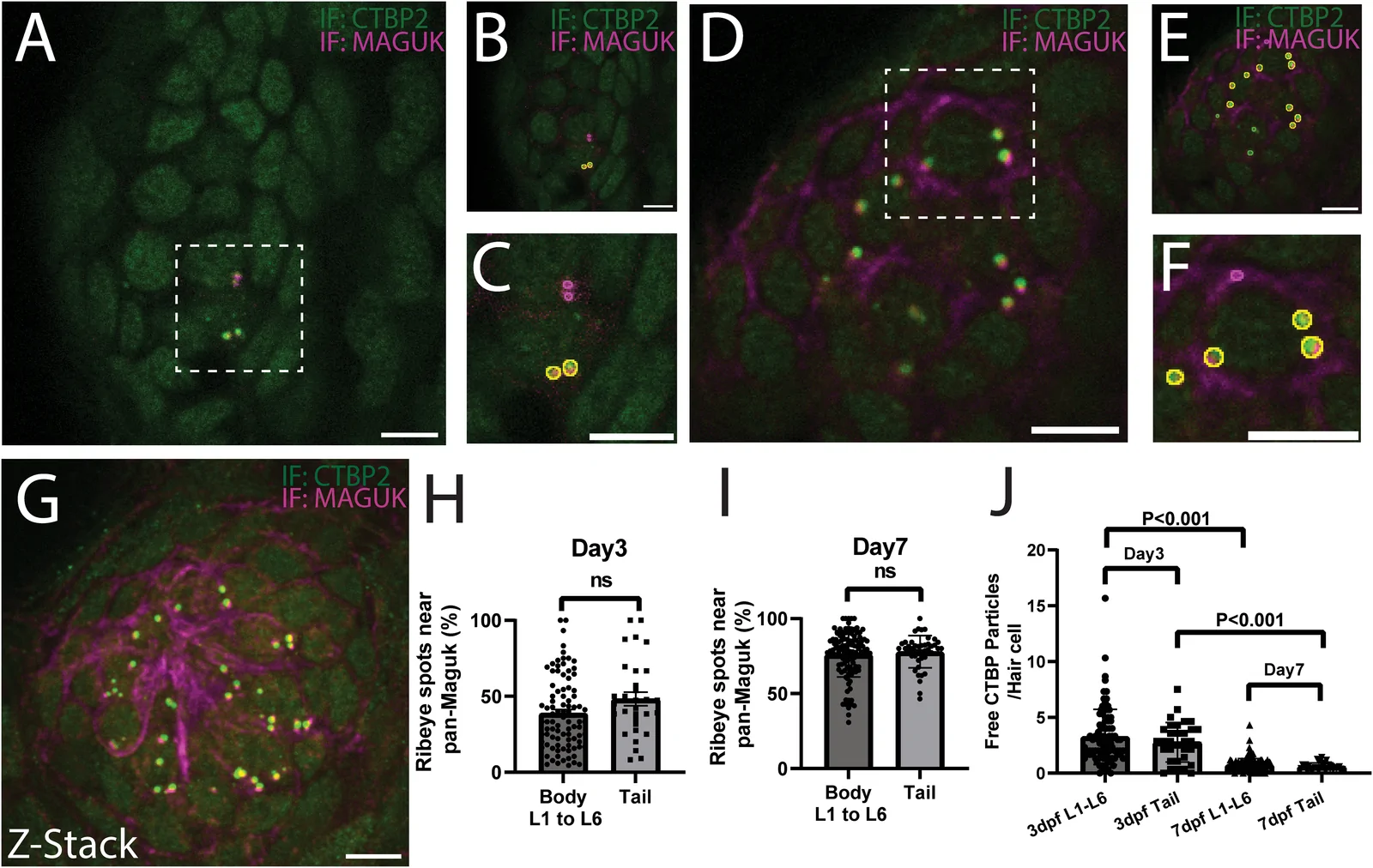
Ribbon synapse development in tail and body. ***A****–**F***, Immunostaining of ribbon markers CTBP2 (green) and postsynaptic marker pan-MAGUK (magenta) in Day 3 (***A****–**C***) and Day 7 (***D****–**F***) animals. Examples of colocalized CTBP2 and pan-MAGUK are outlined in yellow and noncolocalized spots are outlined in magenta (pan-MAGUK) or green (CTBP2). ***B****, **C**, **E**, **F***, Magnified regions from ***A*** and ***D***, respectively. ***G***, *Z*-stack with maximum intensity projection of a Day 7 neuromast across 54 confocal slices covering 12.19 µm. ***H****, **I***, Synapse development, assessed based on the percentage of presynaptic spots that colocalized to postsynaptic spots for 3 dpf (***H***) and 7 dpf (***I***) animals. The number of CtBP/ribeye spots (presynaptic, green) and pan-MAGUK spots (postsynaptic, magenta) per neuromast was counted at L1–L6 and tail locations. A total of 141 hair cells for 3 dpf and 168 hair cells at 7 dpf were analyzed. No significant difference between tail and body was found at 3 dpf (*p* = 0.07) or 7 dpf (*p* = 0.7). ***J***, The number of noncolocalized CTBP2 spots at 3 dpf was 3.3 ± 0.3 at the body, 2.8 ± 0.3 at the tail, and at 7 dpf was 0.7 ± 0.1 at the body and 0.6 ± 0.1 at the tail.

We next examined recruitment of Ribeye to synapses across location and developmental time. For this, we used immunostaining of Ribeye and MAGUK as above, but focused on the fraction of Ribeye spots (marked by anti-CtBP2 antibodies) that colocalized with spots marked by the postsynaptic Maguk (Fig. 3*A*–*G*). There was no difference between the body and tail regions at a given dpf. However, the percentage of Ribeye spots colocalized with postsynaptic Maguk spots nearly doubled from ∼40% at 3 dpf to ∼75% at 7 dpf for both regions (Fig. 3*H*,*I*). Combined with results above, this implies that the number of extrasynaptic Ribeye spots per cell must have decreased from 3 to 7 dpf, reflecting recruitment of Ribeye to synapses. This was indeed the case, as shown in Figure 3*J* (at 3 dpf, 3.3 ± 0.3 at L1–L6 and 2.8 ± 0.3 at tail position; and at 7 dpf, 0.7 ± 0.1 at L1–L6 and 0.6 ± 0.1 at tail position). These results also indicate that while the degree of colocalization of pre- to postsynaptic markers changed dramatically over this period, the synapse number alone cannot account for the differences in capacitance response magnitudes in the tail region relative to the body.

Surprisingly, our results suggest that both synapse number and exocytic capacity remain unchanged during early development, despite the poor localization of Ribeye to pan-MAGUK at early time points and other well-documented changes in properties that hair cells undergo over this period (Santos et al., 2006; Sheets et al., 2011, 2012). It is worth noting that genetic elimination of ribbon synapses does not cause a reduction in capacitance responses in either zebrafish (Lv et al., 2016) or mammalian hair cells (Becker et al., 2018; Jean et al., 2018), consistent with our findings that hair cells that have not finished recruiting Ribeye to assembled ribbon synapses can support normal amounts of exocytosis.

### Otoferlin expression in lateral line hair cells

As an additional test for visualizing hair cells and to assess developmental changes to proteins involved in synaptic transmission, we investigated otoferlin expression. Otoferlin is a key protein required for hair cell exocytosis, which, when mutated, leads to inherited forms of human deafness. To date, 296 mutations and deletions in the otoferlin gene (*Otof*) have been identified, resulting in a spectrum of auditory impairments ranging from mild hearing loss to profound deafness (Pangrsic et al., 2012; Stenson et al., 2020), and otoferlin-deficient mice (*Otof*^−/−^) exhibit profound deafness (Roux et al., 2006). In these mice, exocytosis in inner hair cells and type I vestibular hair cells is nearly abolished despite normal ribbon synapse morphology and Ca^2+^ currents (Roux et al., 2006). These findings underscore otoferlin's essential role in synaptic vesicle exocytosis and suggest it may serve as the primary Ca^2+^ sensor triggering membrane fusion at the inner hair cell ribbon synapse (Dulon et al., 2024).

During mammalian development, inner hair cells undergo a developmental switch that shifts from a mode of neurotransmitter release that is Synaptotagmin-1 dependent to one that requires otoferlin (Reisinger et al., 2011). To determine Otoferlin localization, we first screened several commercially available antibodies in HEK cells transfected with zebrafish Otoferlin A or Otoferlin B for positive signals in transfected cells (Extended Data Fig. 4-1). In our hands, only one of the five antibodies we screened detected Otoferlin A and Otoferlin B, anti-Otoferlin antibody Picoband (PB9616). The others (C-12 from Santa Cruz Biotechnology; 13A9 from Abcam; NBP1-46574 from Novus Biologicals; or HCS-1 from Developmental Studies Hybridoma Bank) did not recognize Otoferlin A or Otoferlin B.

**Figure 4.**
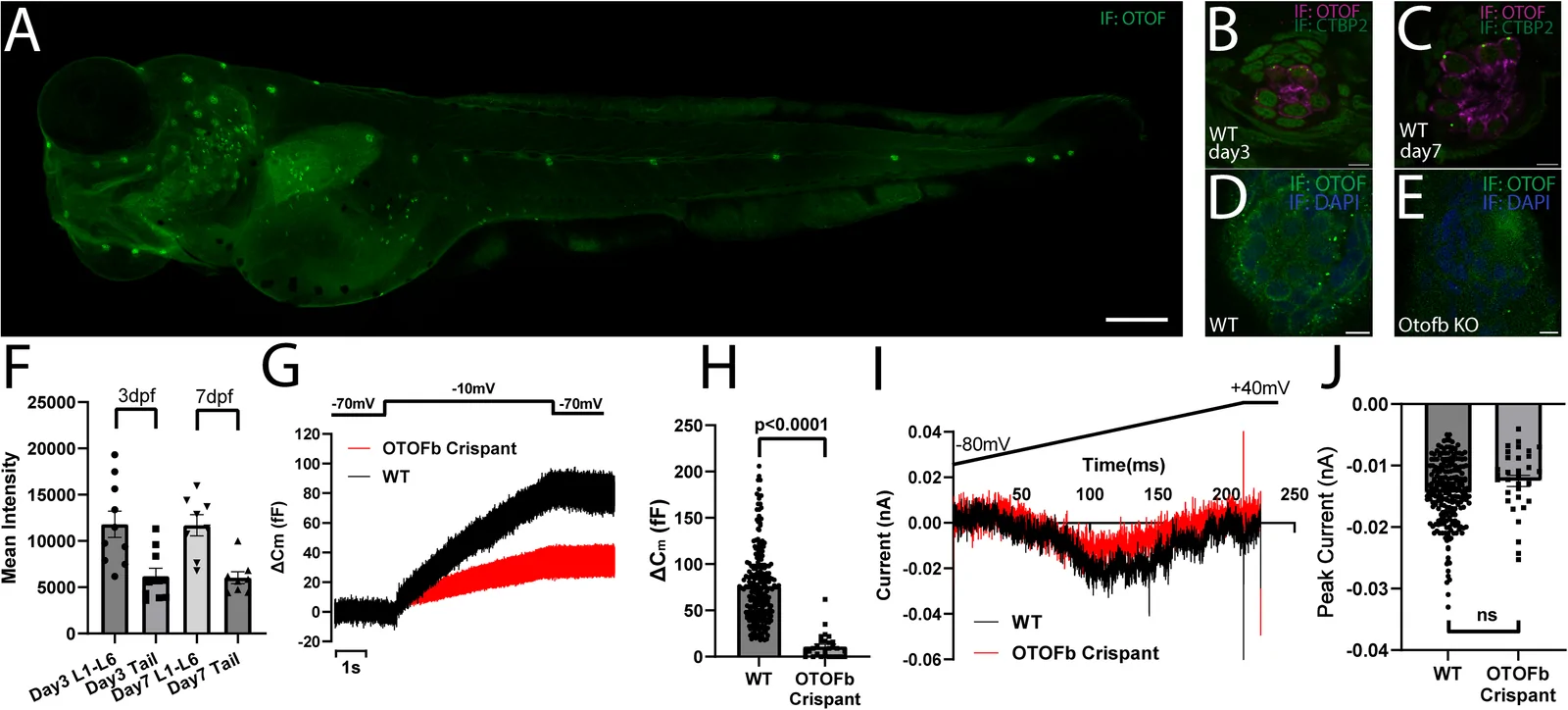
Otoferlin b-mediated exocytosis in zebrafish neuromast hair cells over development. ***A***, Immunostaining for otoferlin (Green) in whole fish at 6 dpf. ***B***, Otoferlin (magenta) and CTBP2 (green) staining of a 3 dpf neuromast. ***C***, Same, for 7 dpf. ***D***, Immunostaining of otoferlin (green) in a neuromast from a WT animal. DAPI (blue) labels nuclei. ***E***, Same as in ***D***, but for *otoferlin b* crispants. ***F***, Otoferlin expression was quantified via immunofluorescence staining in hair cells from the L1–L6 (body) and tail neuromasts at two developmental time points (Day 3 and Day 7). At Day 3, we analyzed 59 neuromasts from the body and 17 neuromasts from the tail across 10 larvae. For Day 7, we quantified expression in 46 neuromasts from the body and 16 neuromasts from the tail across nine larvae. Analysis of the mean fluorescence intensity for each neuromast revealed consistently higher otoferlin expression in hair cells of the L1–L6 region compared with tail hair cells at both Day 3 (*p* = 0.004) and Day 7 (*p* = 0.001). ***G***, Whole-cell capacitance measurements of 7 dpf WT (black) and *otoferlin* crispant (red) zebrafish in response to 5 s depolarizations from −70 to −10 mV. The traces are averages from six recordings each at position L3. ***H***, Average Δ*C_m_* of WT (*n* = 228) and *otofb* gRNA-injected embryos at 5–7 dpf (*n* = 20) neuromast hair cells in response to 5 s depolarizations to −10 mV. The capacitance change was 76.4 ± 2.6 fF (mean ± SEM) for WT hair cells and 15.4 ± 3.0 fF for *otofb* crispants. ***I***, Typical currents in response to a voltage ramp for WT (black) and *otoferlin b* crispant animals (red). ***J***, Average calcium currents are shown for WT (*n* = 231) and *Otofb* (*n* = 32) 7 dpf animals injected with gRNA as embryos, in response to 5 s depolarizations to −10 mV. The inward calcium peak current was −14.4 ± 0.3 pA for WT hair cells and −12.5 ± 0.9 pA for crispants. The antibody was validated in transfected HEK-293 cells (Extended Data Fig. 4-1).

Zebrafish were collected at 3 and 7 dpf and fixed and stained for Otoferlin using the PB9616 antibody. Upon imaging, neuromasts were readily apparent as the most intensely stained structures within the zebrafish, consistent with high expression levels of otoferlin (Fig. 4*A*). Our results indicated no significant differences in intensity between Day 3 and Day 7 at each location (Fig. 4*B*,*C*). Notably, otoferlin staining revealed lower intensity in hair cells located on the tail compared with those on the body, possibly indicating a regional variation in Otoferlin expression and consistent with reduced exocytosis in the tail region (Fig. 4*F*).

10.1523/ENEURO.0479-25.2026.f4-1 Figure 4-1 **Detection of zebrafish otoferlin isoforms by commercially available anti-otoferlin antibodies in HEK293T cells**. Representative confocal images of HEK293T cells transiently transfected with full-length zebrafish otoferlin constructs, either OtofB (OC12: OtofB (Danio rerio)-sg-3Flag) or OtofA (MA8: OtofA (Danio rerio)-sg-3Flag). Cells were transfected using Lipofectamine 2000 according to the manufacturer’s instructions and allowed to express the constructs for 48 h prior to fixation. Anti-Flag immunostaining was used as a positive control to confirm proper expression of the tagged constructs. Nuclei were counterstained with Hoechst (blue). Red fluorescence corresponds to staining obtained with the indicated primary antibodies. Antibodies are shown in order as follows: Otoferlin Antibody (C-12; sc-271092, Santa Cruz Biotechnology), anti-otoferlin [13A9] antibody (Abcam), OTOF Antibody (HCS-1, DSHB), Anti-Otoferlin Antibody Picoband™ (PB9616), and Anti-Flag DYKDDDDK Tag Monoclonal Antibody (FG4R). Scale bars, 10 µm. Download Figure 4-1, TIF file.

### Otoferlin b plays an important role in zebrafish lateral line hair cells

Zebrafish have two otoferlin genes, designated *otoferlin a* and *otoferlin b*. *otoferlin b* (*otofb*) has been shown to be the dominant isoform in neuromasts (Chatterjee et al., 2015). To investigate the role of Otoferlin b in hair cell exocytosis, CRISPR-Cas9 protein was coinjected with two gRNAs targeting exons 39 and 40 of *otofb* at the one-cell stage to generate *otofb* crispants. A subset of crispants was collected for immunostaining to assess Otof protein expression and CRISPR efficiency. As expected, CRISPR-Cas9-injected larvae displayed far less Otoferlin intensity as shown in Figure 4, *D* and *E*.

Additionally, crispants from Days 3–7 were used for in vivo membrane capacitance recordings in neuromast hair cells. *Otofb* crispants showed a dramatic reduction in capacitance response to 5 s depolarizations, as shown in Figure 4*G*. On average, WT hair cells from L1 to L6 exhibited an increase of 76.4 ± 2.6 fF during depolarization, whereas in *otofb* crispants, the response was significantly reduced to 15.4 ± 3.0 fF (Fig. 4*H*; Mann–Whitney, *p* < 0.0001), suggesting the absence of an otoferlin-independent phase of exocytosis during this developmental time period.

We also assessed calcium currents during whole-cell voltage-clamp experiments. To do so, cells were recorded with a solution designed to block most K^+^ currents (see Materials and Methods). An inward Ca^2+^ current was observed during an *I*–*V* ramp protocol (Fig. 4*I*). The average negative Ca^2+^ peak current in WT hair cells was −14.4 ± 0.3 pA. In *otofb* crispants, the Ca^2+^ influx trended smaller by ∼20%, with an average current of −12.5 ± 0.9 pA (Fig. 4*J*), though the diference did not reach statistical significance (*p* = 0.075).

## Discussion

### Hair cell exocytosis development from 3 to 7 dpf

Hair cells located in the neuromasts of the lateral line system are known to play a critical role in detecting water flow and evading predators (Dijkgraaf, 1963; McHenry et al., 2009; Stewart et al., 2013; Higham et al., 2015; Scott et al., 2023). Deflections of stereocilia lead to depolarization and release of glutamate via exocytosis. Here, we examined how these properties change along the axis of zebrafish and during development by collecting electrophysiological data from 3 to 7 dpf fish at different neuromast locations. During this period, hair cells undergo developmental changes (Santos et al., 2006; Sheets et al., 2011, 2012; Kindt et al., 2012; Zhang and Kindt, 2022), suggesting changes in electrophysiological and synaptic properties over that time. Indeed, previous literature indicates that at 3 dpf, neuromasts contain immature hair cells with reduced mechanotransduction (MET) capabilities resulting hair cells from 3 dpf animals displaying reduced afferent firing rates compared with 7 dpf animals (Hardy et al., 2021). We observed no significant differences in exocytosis, probed by capacitance recordings in response to step depolarizations. Our results suggest that the exocytic capacity of neuromast hair cells develops before the maturation of synaptic connections with afferent neurons or that MET may mature over this time. Using pre- and postsynaptic markers to probe synapse development, we found that synaptic connections undergo significant development between Day 3 and Day 7, as the percentage of ribeye spots that localize near postsynaptic markers increases dramatically over this period, consistent with previous reports (Sheets et al., 2012). Interestingly, the number of synapses per cell remains roughly the same at about two per synapse through 3–7 dpf, but the number of extrasynaptic Ribeye spots decreases, reflecting the recruitment of Ribeye to synapses. Our results suggest that the ability to support exocytosis precedes the development of MET and full synapse assembly. The ability of presumably immature ribbons to support high release rates is consistent with previous studies on ribbon synapses lacking ribbons. Removal of Ribeye results in the loss of ribbons, yet some synapses in fish (Lv et al., 2016) and mice (Becker et al., 2018; Jean et al., 2018; Mesnard et al., 2022) continue to exhibit the same high exocytic rates without ribbons, suggesting that ribbons may be dispensable for achieving maximal release rates.

### Zebrafish lateral line as a model for studying exocytosis

Zebrafish larvae present a versatile model for the study of hair cell exocytosis. Its unique advantages including the large numbers of offspring, external development, direct access for in vivo electrophysiology, rapid development, and accessible genetic tools allow for rapid molecular manipulation and functional studies. The rapid generation of CRISPR/Cas9-based crispants within a matter of days permits the efficient evaluation of genetic contributions to exocytotic mechanisms. This combination of physiological and genetic tractability establishes the zebrafish larva as an ideal platform for accelerating the discovery of gene functions regulating vesicular release.

Despite these advantages, rapid developmental changes in the expression of proteins involved in vesicular trafficking may occur and confound interpretation. Our work is a natural extension of the work from previous hair cell studies (Ricci et al., 2013; Olt et al., 2014), which carefully characterized ionic currents and capacitance changes in larval fish (defined as 3–4.5 dpf) and compared them with those of juvenile (29–34 dpf) fish. Previous work found calcium currents to be unchanged between juvenile and larval fish accompanied by increased capacitance responses in the juvenile animals. We find no significant changes in the capacitance responses between 3 and 7 dpf, suggesting that changes in capacitance responses occur later in development.

Hair cells within individual neuromasts have been shown to respond differently, indicating a functional asymmetry based on hair cell orientation. Previous reports have described the differences in hair cells based on their anterior versus posterior location within a single neuromast by calcium imaging of neuromasts (Kindt et al., 2012; Kindig et al., 2023). In our study, we found no significant differences between cells from different neuromasts within the fish, but did not explore differences within single neuromasts. This will be an interesting avenue for future exploration.

While we did not measure MET properties in these experiments, our previous results using the same procedures (Ricci et al., 2013) revealed that MET currents are typically retained in these recordings. However, we cannot be certain that stereocilia were not damaged, and we expect that the tricaine we use to anesthetize fish in these experiments will impact MET currents (Farris et al., 2004). Because the hair cells are voltage-clamped, MET channels are not voltage-activated and are localized at the opposite end of the cell, we do not expect that mechanical currents in the hair bundle influence the capacitance or voltage-gated currents measured in this study.

A protein critical for exocytosis in hair cells is Otoferlin (Azaiez et al., 1993; Yasunaga et al., 1999; Roux et al., 2006). In this study, we tested its expression and requirement in early development in neuromasts. Previous reports in inner hair cells in mammals have demonstrated a developmental (Johnson et al., 2005) switch from a synaptotagmin-dependent process early in development to an Otoferlin-requiring process as hearing onsets after birth (Reisinger et al., 2011). Here, we found that reduction of otoferlin dramatically reduced the exocytic response in both 3 and 7 dpf animals to a similar extent. This suggests that neuromast hair cells in zebrafish, unlike mammalian inner hair cells, either do not pass through an Otoferlin-independent stage or do so prior to 3 dpf. Of note, our crispants continue to exhibit capacitance changes, albeit with smaller amplitudes. This residual exocytosis could arise from either incomplete removal of *otof b* or contribution of other Ca sensors in hair cells. To explore possible candidates that could mediate any residual release, we investigated published single-cell RNAseq data and found few or no transcripts of the synaptotagmins typically associated with fast-exocytosis in developing neuromast hair cells: syt1a, syt1b, or syt2b (Farrell et al., 2018). A further investigation into this published dataset revealed that of the 21 isoforms of synaptotagmin, only Syt 3 and Syt 14b showed detectable levels of expression in lateral line hair cells. Syt3, which was detected at low levels in a minority of cells, is a high-affinity Ca-sensor that underlies vesicle mobility and short-term plasticity in central neurons (Weingarten et al., 2022, 2024). Its low expression level makes it unlikely to significantly contribute to release. Syt14b is expressed at significant levels in hair cells but lacks the ability to bind calcium and thus also unlikely to act as a calcium sensor for release. In contrast, Otof A is consistently expressed in neuromast hair cells, albeit at much lower levels than Otof B.

A previous report found that morpholino knockdown of both *otoferlin a* and *otoferlin b* expression in zebrafish led to changes in ribbon morphology and number, changes in gene expression, increased basal calcium concentration, and reduced fluorescence uptake of a membrane-attached dye, reflecting a reduction in membrane turnover (Manchanda et al., 2019). We found that reduction in expression of Otof b alone via CRISPR/Cas9 is sufficient to dramatically reduce overall exocytosis, consistent with its dominant expression in neuromasts (Farrell et al., 2018). It seems unlikely that the modest reduction in ribbon number or change in ribbon morphology contributes significantly to the dramatic reduction in capacitance response, since dramatic or complete reduction in synaptic ribbons leads to modest or no changes in depolarization-driven calcium responses. We did not measure basal calcium levels in our study. An increase in basal calcium would be expected to enhance release and could partially mask the magnitude of the effect of Otof b removal. We found no effect on Ca^2+^-current magnitude with Otof b removal. Though the small currents could preclude discovery of a small, yet important change, our results are consistent with the normal calcium responses observed in morphants in response to mechanical stimulation. Together, these results support the idea that basal calcium levels are downstream of changes in resting potential and/or the previously reported changes in expression of calcium-binding proteins (Manchanda et al., 2019).

### Distinct functions and patterns of tail neuromasts

Based on anatomy, some studies consider that there are five primary lateral and three terminal (tail) neuromasts (Ghysen and Dambly-Chaudiere, 2007; Ma and Raible, 2009), while others (Holmgren and Sheets, 2020) report six primary lateral and two terminal ones. Our recordings revealed differences in the capacitance responses to depolarization for hair cells localized to the two tail neuromasts in comparison with the six others on the body: exocytosis was reduced by ∼40% during depolarization in the tail region. Thus, our studies provide a functional basis for the classification of neuromasts as six primary (L1–L6) and two tail (Tail1–Tail2) neuromasts. The functional differences we observed between the hair cells belonging to these neuromast classes may reflect distinct biological roles and adaptations to the different mechanical sensory environments of each neuromast region. Specifically, neuromasts L1–L6 are strategically positioned to detect water flow from forward and backward directions, a function critical for spatial orientation and predator detection (McHenry et al., 2009). Hair cells in the body, particularly those near the head and trunk, are often more exposed to subtle environmental changes than the tail. Because exocytosis is proportional to stereocilia deflection (Trapani and Nicolson, 2010; Kindt et al., 2012), smaller stereocilia deflections in the body may couple with higher exocytotic output to ensure reliable signaling. In contrast, the tail's primary function is thrust generation, resulting in larger stereocilia deflections. The reduced exocytotic output we observed in tail hair cells could prevent signal saturation, effectively “turning down the volume” to maintain the range of signaling for tail-generated stimuli. Nevertheless, our data show that synaptic density per hair cell remains consistent. These results emphasize that studies of electrophysiological and synaptic properties of zebrafish hair cells should make note of these differences and account for them when interpreting their results. Furthermore, these results imply that structural and/or molecular aspects in the tail region may differ from those in the body, including differences in calcium currents and otoferlin expression. Furthermore, differences and mechanisms remain to be elucidated.

## Synthesis

Reviewing Editor: Matthew Grubb, King's College London Decisions are customarily a result of the Reviewing Editor and the peer reviewers coming together and discussing their recommendations until a consensus is reached. When revisions are invited, a fact-based synthesis statement explaining their decision and outlining what is needed to prepare a revision will be listed below. The following reviewer(s) agreed to reveal their identity: NONE. Note: If this manuscript was transferred from JNeurosci and a decision was made to accept the manuscript without peer review, a synthesis may not be available.

Synthesis:

Both Reviewers request several revisions to the manuscript, and we expect all such points to be fully addressed in the re-submission (see reviews below).

Please note that the potential lack of novelty identified by Reviewer 2 does not impact on suitability for publication in eNeuro. There is no need to respond to this point.

Reviewer 1:

In their manuscript, Electrophysiological profiling of exocytosis during early-stage development of the zebrafish lateral line, the authors examine synaptic vesicle release (as determined by voltage-clamp capacitance measurements) in larval hair cells across early development (day 3 to day 7 post fertilization) in the posterior lateral line at locations along the trunk and tail. Comparing their data with immunohistochemistry of pre- and post-synaptic markers, they find that while the ribbon synapses of developing hair cells undergo maturation and refinement during this time, peak capacitance measurements in response to 5-second voltage steps do not change. The authors also found that hair cells of tail neuromasts appear to maintain lower rates of release, which may have functional benefits given the different type of forces detected by tail neuromasts compared to trunk neuromasts (as mentioned in the Discussion). Finally, the majority of the patch-clamp evoked vesicle fusion is shown to rely on the synaptic protein otoferlin, which is similar to findings from mammalian hair cells though in the latter, synaptotagmin 1 is used for pre-hearing hair cell function. Overall the manuscript is well written and the data are sound and will benefit the field in its understanding of hair cell function in the zebrafish lateral line as well as serve as a baseline for future work examining the role of synaptic proteins in exocytosis using the larva zebrafish model system. Below are some specific requests that will strengthen the article and a few minor points to be addressed.

**Specific requests to address:**

Introduction and Discussion: Capacitance measurements from Olt et al 2014 (figure 8) are worth citation and mention. In that study, the authors show a steady-state capacitance baseline, which would be beneficial to implement in all capacitance traces in the figures here. Also their measurements show that hair-cell capacitance had reached a maximum by the first second of depolarization and then held constant, please discuss in comparison to the current study which shows a steady rising capacitance over 5 seconds (which suggests retrieval rate might not be able to keep up with fusion) in the current study.

Introduction: The manuscript does not fully investigate/analyze (nor show) voltage-gated calcium currents in a meaningful way and perhaps the first sentence of the 4th paragraph of Introduction should be amended to better reflect this absence (as does the Abstract).

The third paragraph of the Results mentions "quantification" of capacitance. Quantification is not described in the Methods section. How was delta Cm determined, was it compared to a baseline Cm (note: there is no Cm raw data shown outside of during the voltage step, which would be ideal to show). In addition, the rate of change of Cm over time appears to be similar across days, which would strengthen the argument that there is no difference in fusion rates during this time period. Examples of the fits could be shown with the raw data in Fig.1B,C,D and 4G, which would strengthen the quantitative nature of the authors conclusions.

Figures: Raw data traces from patch-clamp currents should go along with Figure 1G. In Figure 4, raw traces for an I-V ramp are shown but the analyzed data are from an I-V step protocol and showing those raw voltage-clamp data to go with 4J (in addition to or instead of the ramp data) is warranted.

Discussion: Given the potential differences in hair cells within a neuromast (Williams &Holder, 2000; López-Schier &Hudspeth, 2006; Kindt et al. 2012), and the finding of Kindig et al., 2025 where there are functional differences in hair cells that are anterior versus posterior. Please mention which hair cells (location within a neuromast) were typically patched in Methods as whether the patched hair cell's location was always from the same region within a neuromast or whether it varied would be interesting when considering the results in the context of the above studies.

Discussion: Is it known whether the mechanotransduction properties (incl. intact hair bundles) of the patched hair cells are retained (see Olt et al., 2014)? If not, the manuscript would benefit from mention of how this may or may not impact the data from voltage-clamp induced SV fusion.

**Minor points to address:**

Accessibility: Panels in Figures 2, 3, and 4 with green and red colors would benefit from changing to green and magenta/purple for individuals with red/green color blindness.

Paragraph 2 of the Introduction and 4 of the Results are quite short.

Second paragraph of Results: there are some redundant sentences.

Second paragraph of Results section: there are likely more than just 3 secondary neuromasts deposited by Prim2 (see Ghysen 2007). Also, some studies state that there are 5 primary neuromasts and 3 terminal (e.g., Ghysen 2007 and Ma and Raible 2009), which might be interesting to mention and then discuss as your 6th primary NM data may serve to show that it is better to consider it a primary and that indeed there are just two terminal NMs as defined functionally by your capacitance data.

Figure 2H: the data points (horizontal lines) for some of the plots do not look correct, if made in Graphpad Prism maybe choose "expanded" for dot plot style.

Figures 2B, 2C, there are green circles around non-colocalized spots that are not described in the legend as they are in Figure 3.

Figure 4: There is text within [square brackets] that appears to be an artifact from someone reviewing the text in the legend.

For Figure 4J and others, the Methods section states "Student's t test or ANOVA" tests were performed, but no mention of whether normality tests were done to determine which was chosen.

Reviewer 2:

The study by Wang et al. reports the results of electrophsiology and microscopy conducted on zebrafish neuromasts. The quality of the data provided is reasonable, however the study provides little to advance the field. Addressing the concerns below would improve the study.

1) Exocytosis appears otoferlin dependent at all ages, which distinguishes it from mouse hair cells which switch from synaptotagmin to otoferlin. The authors should test whether synaptotagmin is expressed in zebrafish hair cells. This would be informative.

2) The authors report calcium currents in otob crispants but not resting Ca2+, which was reported in Manchanda et al. 2019. Manchanda et al. also investigated the influence of otoferlin on ribbon development and gene expression, which do not appear to be discussed in this manuscript. In fact I cannot find any reference to the 2019 paper, which should be referenced given that it focuses on the same topic as this current study.

3) Does oto a upregulate or express in compensation for the loss of otob? Are VGlut3 or ribeye expression levels altered? There is little characterization of the synapse reported for the otob crispants

4) Was the otoferlin antibody used in this study tested to ensure no off-target interactions? Does it react with otoa in WT and otob crispants?

5) Otoferlin b is mentioned several times before an explanation of it being one of two genes is provided. The authors may want to define otoferlin b earlier in the mansucript.

## Author Response

We thank the reviewers for their thoughtful and constructive feedback, which has helped us to significantly strengthen the manuscript. We have carefully considered all comments and have prepared a point-by-point response detailing the revisions made to address each concern.

Reviewer 1:

1. "Introduction and Discussion: Capacitance measurements from Olt et al 2014 (figure 8) are worth citation and mention. In that study, the authors show a steady-state capacitance baseline, which would be beneficial to implement in all capacitance traces in the figures here. Also their measurements show that hair-cell capacitance had reached a maximum by the first second of depolarization and then held constant, please discuss in comparison to the current study which shows a steady rising capacitance over 5 seconds (which suggests retrieval rate might not be able to keep up with fusion) in the current study." We regret the oversight of not referencing Olt et al., 2014 or Olt et al., 2016 in our original submission and thank the reviewer for bringing it to our attention. These references have been added to the appropriate places in the Results and Discussion sections, and we have included the baseline, as requested. However, figure 8 shows a response from a single time point and does not indicate whether capacitance reaches a plateau at that time point. From re-reading their papers, we could not find any data that addresses the magnitude of capacitance change with increasing depolarization duration.

2. "Introduction: The manuscript does not fully investigate/analyze (nor show) voltage-gated calcium currents in a meaningful way and perhaps the first sentence of the 4th paragraph of Introduction should be amended to better reflect this absence (as does the Abstract)." We agree to an extent with the reviewer here that we did not fully investigate the currents, but we did measure inward currents that are presumed calcium currents (Figure 1H, I) and found no significant variation across time, but with significant differences in the tail and body. We have tempered some of our discussion in the introduction and removed mention of the calcium currents in the abstract.

3. "The third paragraph of the Results mentions "quantification" of capacitance. Quantification is not described in the Methods section. How was delta Cm determined, was it compared to a baseline Cm (note: there is no Cm raw data shown outside of during the voltage step, which would be ideal to show). In addition, the rate of change of Cm over time appears to be similar across days, which would strengthen the argument that there is no difference in fusion rates during this time period".

We have added a description of our capacitance quantification method in Materials and Methods, explaining how delta Cm was determined relative to a pre-step baseline, now we add base line before the depolarization in Figure 1(B-D) and Figure 4(G). As suggested by the reviewer, we have now fit each trace to a single exponential and report the average time constant across developmental time. We find no differences in the rates of release.

4. "Examples of the fits could be shown with the raw data in Fig.1B,C,D and 4G, which would strengthen the quantitative nature of the authors conclusions." We thank the reviewers for their suggestions and have re-analyzed the capacitance recording traces at 3, 5, and 7 dpf. We fit individual depolarizations using a single exponential function: C=A(1-exp⁡(-(t-t_0 )\/τ) )+b. A representative capacitance recording at 7 dpf is shown above (A), along with the corresponding fitted curve in red; the average τ values are presented in (B). From this τ analysis, we did not observe significant differences. We have also incorporated this finding into the Results section 5. "Raw data traces from patch-clamp currents should go along with Figure 1G." We have added current traces in response to voltage ramps in Fig1H in the revision.

6. "In Figure 4, raw traces for an IV ramp are shown but the analyzed data are from an I-V step protocol" The calcium current magnitudes were determined by IV-ramps, not the steps. We have corrected the manuscript to state this. We apologize for our mistake. The data analyzed in Figure 4J now correctly come from the I-V ramp protocol, we revised the manuscript to ensure clarity.

7. "Discussion: Given the potential differences in hair cells within a neuromast (Williams &Holder, 2000; López-Schier &Hudspeth, 2006; Kindt et al. 2012), and the finding of Kindig et al., 2025 where there are functional differences in hair cells that are anterior versus posterior. Please mention which hair cells (location within a neuromast) were typically patched in Methods as whether the patched hair cell's location was always from the same region within a neuromast or whether it varied would be interesting when considering the results in the context of the above studies." We thank the reviewer for raising this important point about hair cell heterogeneity. As noted in the revised Methods section, hair cells were patched randomly from different regions of the neuromast. We did not distinguish the two groups. These details have now been included in the Methods and discussion 8. "Is it known whether the mechanotransduction properties (incl. intact hair bundles) of the patched hair cells are retained (see Olt et al., 2014)? If not, the manuscript would benefit from mention of how this may or may not impact the data from voltage-clamp induced SV fusion." We have added the following to the manuscript to address this issue: "While we did not measure mechanotransduction properties in these experiments, our previous results using the same procedures (Ricci et al., 2013) revealed that mechanotransduction (MET) currents are typically retained in these recordings. However, we cannot be certain that stereocilia were not damaged and we expect that the tricaine we use to anesthetize fish in these experiments will impact MET currents (Farris et al., 2004). Nonetheless, because these cells are voltage clamped, MET currents are not voltage activated and are located at the opposite end of the cell, we do not expect that mechanically currents in the hair bundle would impact capacitance or volage-gated currents that we measured in this study." Minor:

9. "Accessibility: Panels in Figures 2, 3, and 4 with green and red colors would benefit from changing to green and magenta/purple for individuals with red/green color blindness." Thank you for raising this accessibility issue. We changed the colors as suggested.

10. "Paragraph 2 of the Introduction and 4 of the Results are quite short." We have expanded the second section of the introduction. In the updated version, the fourth paragraph of the results has been revised and merged with the preceding paragraph 11."Second paragraph of Results: there are some redundant sentences." We thank reviewer for pointing this out. We have removed these redundancies.

12. "Second paragraph of Results section: there are likely more than just 3 secondary neuromasts deposited by Prim2 (see Ghysen 2007). Also, some studies state that there are 5 primary neuromasts and 3 terminal (e.g., Ghysen 2007 and Ma and Raible 2009), which might be interesting to mention and then discuss as your 6th primary NM data may serve to show that it is better to consider it a primary and that indeed there are just two terminal NMs as defined functionally by your capacitance data." We acknowledge that the relevant literature (Ghysen, 2007; Ma and Raible, 2009) describes a configuration of three terminal neuromasts in some cases. Nevertheless, in our experimental system, the majority of zebrafish consistently presented with a "6th primary" and two terminal neuromasts. We have added references to the Results section, as these findings provide support for the classification employed throughout our manuscript (Ghysen and Dambly-Chaudiere, 2004; Ma and Raible, 2009).

13. " Figure 2H: the data points (horizontal lines) for some of the plots do not look correct, if made in Graphpad Prism, maybe choose "expanded" for dot plot style" .

Thank you for your comment. We have corrected this in the revision. Thank you for your comment.

14. "Figures 2B, 2C, there are green circles around non-colocalized spots that are not described in the legend as they are in Figure 3." The green circles in Fig. 2B and 2C indicate non-colocalized spots with the Maguk staining spot. We have now revised the description in the figure legends accordingly.

15. "Figure 4: There is text within [square brackets] that appears to be an artifact from someone reviewing the text in the legend." We thank the reviewer for pointing out this mistake, which has now been removed.

16. "For Figure 4J and others, the Methods section states "Student's t test or ANOVA" tests were performed, but no mention of whether normality tests were done to determine which was chosen" Thank you for raising this important statistical point. We performed Lilliefors and Jarque-Bera normality tests, for which our data failed. All comparisons are performed using the Mann-Whitney test, which is appropriate for skewed non-normal samples.

Reviewer2 1. "Exocytosis appears otoferlin dependent at all ages, which distinguishes it from mouse hair cells which switch from synaptotagmin to otoferlin. The authors should test whether synaptotagmin is expressed in zebrafish hair cells. This would be informative." To address these questions, we looked at published single-cell RNAseq data (Farrell et al., 2018) and have added it to the discussion of this paper. In short, there is little to no expression of the calcium-dependent synaptotagmins in hair cells over this period. Developing neuromast hair cells show no or very few transcripts for syt1a and syt1b and syt2a. Some expression of Syt3 and syt7a show expression after 72hpf ,whereas Syt9b expressed after 120hpf. None of the Ca²⁺-dependent Syts show strong transcription during early development, whereas otofb shows the strongest expression after 48 hpf, which aligns with the lateral line development timeline. This strongly suggests a difference between zebrafish and mammals. Furthermore, Otof crispants strongly reduce Ca2+-dependent exocytosis, which establishes that the major Ca2+ sensor is Otof. We have included this in our revised discussion.

2. "The authors report calcium currents in otob crispants but not resting Ca2+, which was reported in Manchanda et al. 2019. Manchanda et al. also investigated the influence of Otoferlin on ribbon development and gene expression, which do not appear to be discussed in this manuscript".

We thank the reviewer for pointing this out. The Manchanda paper is indeed relevant here and we apologize for the oversight. We did not measure resting Ca2+ in our paper, but we now discuss the implications of our calcium current measurements to the observations of Manchanda. We also discuss the implications of the ribbon development and gene expression findings in the interpretation of our results. These can be found in the new discussion.

3. "Does otof a upregulate or express in compensation for the loss of otob? Are VGlut3 or ribeye expression levels altered? There is little characterization of the synapse reported for the otob crispants" While we have not investigated the question of changes in OtofA compensation, we view it unlikely to be significant for two reasons: 1) exocytosis is greatly reduced with the removal of OtofB. If there is an upregulation of OtofA in these cells, then it is apparently insufficient to rescue function; 2) Our antibody was raised against C-terminal of human otoferlin and does not distinguish between OtofA and OtofB in zebrafish (new supplementary figure). We find labeling nearly absent in lateral line hair cells in crispants.

As for vGLUT3, we have not performed IHC or qPCR for VGlut3, but in a previous report by Chatterjee et al., disruption of otoferlin resulted in no changes in Vglut3 expression by qPCR (Chatterjee et al., 2015).

Regarding ribbon synapses, while we did perform IHC on ribeye in some otofb crispants, we did not systematically analyze the size shape and number of ribbons across multiple animals, as done in Manchanda et al.. We did not observe dramatic changes in ribbon morphology, but acknowledge that a more systematic approach investigating changes across multiple animals would be necessary to reach a firm conclusion.

4. "Was the otoferlin antibody used in this study tested to ensure no off-target interactions? Does it react with otoa in WT and otob crispants?" The antibody targets the C-terminal region of the human otoferlin protein. This C-terminal region is highly conserved and shows strong sequence identity with both zebrafish otoferlin a and b. We have tested the antibody in HEK cells transfected with Otof B and Otof A and found specific staining or both. This data is now included as a supplemental figure.

5. "Otoferlin b is mentioned several times before an explanation of it being one of two genes is provided. The authors may want to define otoferlin b earlier in the mansucript." We have moved the definition and explanation of otoferlin a and b in zebrafish to the fourth paragraph of the Introduction.

References Chatterjee P, Padmanarayana M, Abdullah N, Holman CL, LaDu J, Tanguay RL, Johnson CP (2015) Otoferlin deficiency in zebrafish results in defects in balance and hearing: rescue of the balance and hearing phenotype with full-length and truncated forms of mouse otoferlin. Mol Cell Biol 35:1043-1054.

Farrell JA, Wang Y, Riesenfeld SJ, Shekhar K, Regev A, Schier AF (2018) Single-cell reconstruction of developmental trajectories during zebrafish embryogenesis. Science 360.

Ghysen A, Dambly-Chaudiere C (2004) Development of the zebrafish lateral line. Curr Opin Neurobiol 14:67-73.

Ma EY, Raible DW (2009) Signaling pathways regulating zebrafish lateral line development. Curr Biol 19:R381-386.
